# Prognostic significance of CAD-RADS for patients with suspected coronary artery disease: A systematic review and meta-analysis

**DOI:** 10.1093/radadv/umae007

**Published:** 2024-04-01

**Authors:** Shingo Kato, Mai Azuma, Nobuyuki Horita, Daisuke Utsunomiya

**Affiliations:** Department of Diagnostic Radiology, Yokohama City University Graduate School of Medicine, Yokohama 236-0004, Japan; Department of Cardiology, Kanagawa Cardiovascular and Respiratory Center, Yokohama 236-0051, Japan; Chemotherapy Center, Yokohama City University Graduate School of Medicine, Yokohama 236-0004, Japan; Department of Diagnostic Radiology, Yokohama City University Graduate School of Medicine, Yokohama 236-0004, Japan

**Keywords:** CAD-RADS, coronary computed tomography angiography, prognostic value, meta-analysis

## Abstract

**Background:**

Coronary Artery Disease-Reporting and Data System (CAD-RADS) is a standardized reporting system that offers a structured method for interpreting and reporting results obtained through coronary computed tomography angiography. It has been deemed useful in the prognostication of patients with suspected coronary artery disease (CAD).

**Purpose:**

The present meta-analysis sought to assess the prognostic value of CAD-RADS in individuals with suspected CAD.

**Materials and Methods:**

We conducted a systematic search of the electronic databases of PubMed, Web of Science Core Collection, Cochrane advanced search, and EMBASE. A random-effects model was implemented to evaluate the pooled hazard ratio (HR) for each CAD-RADS category and area under the receiver operating characteristics curve for predicting major adverse cardiovascular events.

**Results:**

Data from 37 596 coronary computed tomography angiography examinations from 13 studies were analyzed. During follow-up, 2,536 (6.7%) adverse events were observed. Pooled HRs for prediction of adverse events were significant for all CAD-RADS categories, with incremental increase in HRs with progressively higher categories. For prediction of all-cause mortality, all categories except CAD-RADS 1 showed significant HR compared with CAD-RADS 0. Combination of CAD-RADS to conventional clinical risk factors and CAC resulted in a high predictive capacity for adverse events (pooled area under the receiver operating characteristics curve: 0.82 ([95% confidence interval, 0.73-0.91]).

**Conclusion:**

The CAD-RADS category imparts information on the CAD severity and shows incremental increase in HR for adverse events with progressively higher categories, especially beyond CAD-RADS4b.

**Abbreviations**

AI, artificial intelligence; AUC, area under the characteristic curve; CAC, coronary artery calcium; CAD, coronary artery disease; CAD-RADS, Coronary Artery Disease-Reporting and Data System; CI, confidence interval; CTA, computed tomography angiography; HR, hazard ratio; MACE, major adverse cardiovascular event

**Summary**

The CAD-RADS provides crucial prognostic insights for suspected coronary artery disease, significantly predicting major cardiovascular events and all-cause mortality, especially when combined with clinical risk factors and coronary calcium scores.

**Key results**

•  Coronary Artery Disease-Reporting and Data System (CAD-RADS) presents valuable prognostic information for patients with suspected coronary artery disease, with incremental increase in hazard radios for major adverse cardiovascular events with progressively higher categories.

•  For prediction of all-cause mortality, all categories except CAD-RADS 1 showed significant hazard ratio compared with CAD-RADS 0.

•  Combination of CAD-RADS with conventional clinical risk factors and coronary artery calcium score resulted in a high predictive capacity for adverse events (pooled area under the receiver operating characteristics curve, 0.82 ([95% confidence interval, 0.73-0.91).

## Introduction

Coronary computed tomography angiography (CTA) is widely used globally as a noninvasive imaging modality for ruling out coronary artery disease (CAD).^1^^,^^2^ The 2021 American Heart Association/American College of Cardiology/American Society of Echocardiography (ASE)/American College of Chest Physicians (CHEST)/Society for Academic Emergency Medicine/Society of Cardiovascular Computed Tomography/Society for Cardiovascular Magnetic Resonance guidelines for the evaluation and diagnosis of chest pain recommend coronary CTA as Class 1, Level of Evidence A for patients with stable and acute chest pain with low to intermediate risk of pretest probability.^3^ Coronary CTA enables the visualization of the coronary vessel lumen along with providing information regarding the vessel wall characteristics including remodeling, plaque, thickness, and degree of calcification.^4^^,^^5^ The supplementary information about the coronary artery wall offered by coronary CTA is not available with invasive catheter coronary angiography.^6^ The vast amount of information available from coronary CTA renders it an exceptionally valuable tool for prognostic prediction.^7^^,^^8^

Coronary Artery Disease-Reporting and Data System (CAD-RADS) is a standardized reporting system that provides a structured approach for interpreting and reporting results of coronary CTA.^9^ CAD-RADS is designed to standardize the reporting of the severity of CAD and facilitate communication among healthcare professionals.^10^ The reproducibility of CAD-RADS scores has been found to be moderate to high, with good inter-rater agreement.^11^ Interestingly, several studies have reported that CAD-RADS provides accurate prognostic information about patients with CAD.^12–16^ A systematic review and meta-analysis regarding the prognostic value of CAD-RADS has been published previously.^17^ Interestingly the study revealed no difference between the pooled hazard ratios (HR) of CAD-RADS 4a and 4b, which clearly differ in clinical severity. Furthermore, the incremental clinical risk and prognostic value of CAD-RADS on coronary artery calcium score (CAC) on coronary computed tomography (CT) has not been assessed.

To address these clinical questions, we performed a comprehensive systematic review and meta-analysis evaluating the prognostic significance of CAD-RADS in patients with suspected CAD.

## Materials and methods

### Search strategies and selection criteria

We employed the search methodologies recommended by the Cochrane Collaboration and complied with the Preferred Reporting Items for Systematic Review and Meta-analysis 2020 guidelines for reporting.^18^ On April 21, 2023, we conducted a systematic search using PubMed, Web of Science Core Collection, Cochrane advanced search, and EMBASE electronic databases. The literature search was conducted by S.K. (10 years' experience as a cardiologist and 6 years' experience as a radiologist) and M.A. (10 years' experience as a cardiologist). The search was performed using the following keywords: Coronary Artery Disease Reporting and Data System, CAD-RADS, coronary computed tomography, coronary computed tomography angiography, coronary artery disease and stable angina pectoris (Table S1). The eligibility criteria were restricted to articles that examined the prognostic value of CAD-RADS in patients with CAD and excluded case reports, animal studies, and non-English articles. Two reviewers (S.K. and M.A.) screened all titles and abstracts in the search results, and full texts of any potentially relevant studies were reviewed for eligibility. Any discrepancies were resolved by a third reviewer. The study protocol was registered with the University Medical Information Network (registration number: UMIN000050988). Institutional review board approval was not required because this study was a meta-analysis and did not involve clinical patient information.

### Outcome measures

The primary outcome of this meta-analysis was the pooled HR of CAD-RADS categories in patients with suspected CAD. Two reviewers (S.K. and M.A.) extracted the data regarding the HRs of different CAD-RADS categories (categories 1-5) compared with category = 0 for prediction of major adverse cardiovascular events (MACE) and all-cause mortality, wherever provided, from the included articles. Furthermore, we calculated incremental increase in area under the receiver operating characteristics curve (AUC) for prediction of MACE with addition of CAD-RADS to the traditional clinical risk factors or CAC score from articles where said data was available. Data extraction was carried out using standardized forms.

### Assessment of risk of bias

To assess the risk of bias, we used the Newcastle-Ottawa Quality Assessment Scale and case-control studies.^19^ Additionally, we presented funnel plots for HRs in each CAD-RADS category to assess publication bias.

### Data integration and statistical analysis

We conducted a meta-analysis using RevMan 5.41 (Cochrane Collaboration, London)^20^ methods with random-effects models to estimate the HRs of the pooled CAD-RADS and to assess the incremental prognostic value of the CAD-RADS. We employed chi-square tests to compare HRs for each CAD-RADS. We used the Begg test as a statistical test to quantify publication bias. Heterogeneity was expressed by *I*^2^, where 0% indicates no heterogeneity and 100% indicates strong heterogeneity.^21^ Sensitivity analysis was conducted by adding an analysis using the fixed effect model. For the Begg test, *P* < .10 was considered statistically significant, and for other statistical methods, *P* < .05 was considered statistically significant.

## Results

**Figure 1.**
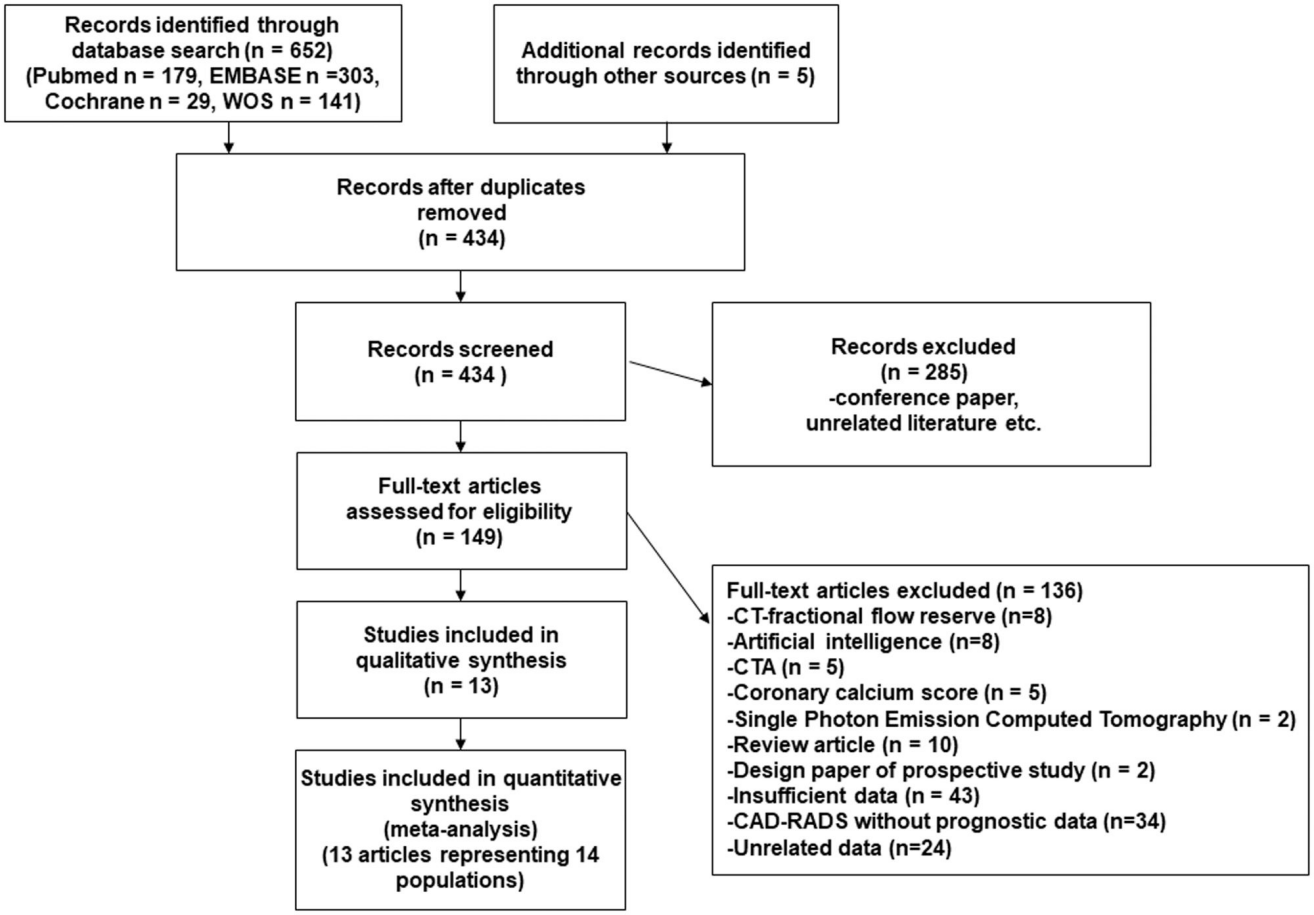
PRISMA flow diagram.

From 432 articles found in the preliminary literature search, we finally included 13 eligible articles (Figure 1),^12–16^^,^^20^^,^^22–28^ and their characteristics are summarized in Table 1. These 13 papers presented data on 14 populations including 37 596 patients. The selected studies were published between 2018 and 2021. Three studies were from the USA^12^^,^^13^^,^^23^ and South Korea,^16^^,^^22^^,^^26^ respectively, whereas 2 were from Germany,^15^^,^^27^ 1 each from Turkey,^24^ China,^25^ the Netherlands,^14^ Austria,^28^ and the United Kingdom.^20^ The study designs included 5 retrospective analyses of large registry studies,^12^^,^^14^^,^^15^^,^^20^^,^^23^ 4 retrospective single-center studies,^13^^,^^22^^,^^25–28^ and 2 retrospective multicenter studies.^16^^,^^24^ The CT scanners used were 64-slice or higher or dual-source CT scanners. Endpoint definitions varied from study to study and are summarized in Table 2.

**Table 1.** Characteristics of included studies.

Name of cohort	Study design	Country	Inclusion criteria	No. of patients	Age, y	male, ％	BMI	Hypertension, %	Diabetes, %	Dyslipidemia, %	Smoking, %	Family history of CAD, %
Altay_2021	Retrospective multicenter	Turkey	Patients underwent CTA	359	Mean: 52 years old in females, mean: 56 years old in males	54.4	N/A	47	11	N/A	73	45
Bittner_2020	Retrospective analysis of PROMISE trial	Germany	Symptomatic patients suspected for CAD	3840	60.4 ± 8.2	48.7	30.3 ± 5.8	64.1	20.3	67.4	51.5	33.2
Finck_2019	Retrospective single	Germany	Suspected CAD (not previously diagnosed)	2011	59.2 ± 11	66	26.1 ± 4.01	58.8	7.3	51.7	20	31.2
Huang_2021	Retrospective single	China	Suspected CAD patients who underwent coronary CTA	9625	59.8 (10.7)	44.3	N/A	N/A	N/A	N/A	N/A	N/A
Johnson_2019	Retrospective single	USA	Patients underwent coronary CTA	6892	N/A	64.6	28.7 (26.3–31.6) for men, 27.5 (23.9–32.1) for women	56.5 for men, 59.2 for women	10.0 for men, 11.8 for women	N/A	12.8 for men, 16.5 for women	24.1 for men, 29.5 for women
Lee_2021	Retrospective multicenter	Korea	Patients with low- to intermediate-risk of CAD (acute chest pain)	1492	58 ±14	51	24 ±3	33	13	14	24	3
Nam_2019	Retrospective single	Korea	Patients with ischemic stroke underwent coronary CTA	762	66 (55, 73)	60.4	23.8 (22.1, 25.9)	58	23.7	32.5	24.1	N/A
Park_2021	Retrospective single center	Korea	Patients with acute chest pain underwent coronary CTA	779	55.8 ± 14.5	53.2	24.1 ± 3.5	22.5	9.6	12.1	13.3	4.7
Senoner_2020	Retrospective single center	Austria	Patients referred for coronary CTA for all clinical indications	1430	57.9 ± 11.1	55.6	26.7 ± 4.4	63.6	13.0	63.5	39.3	38.0
van den Hoogen_2020_DM	Retrospective analysis of LUMC and CONFIRM registry	USA	DM	732	58 ± 12	61	28.3 ± 5.1	63	N/A	58	24	36
van den Hoogen_2020_nonDM	Retrospective analysis of LUMC and CONFIRM registry	USA	Non-DM	732	58 ± 13	60	27.7 ± 5.1	61	N/A	51	23	35
van Rosendael_2019	Retrospective analysis of LUMC and CONFIRM registry	The Netherlands	Patients with suspected but without known CAD	2134	55±13	N/A	N/A	N/A	N/A	N/A	N/A	N/A
Williams_2020	Retrospective analysis of SCOT-HEART trial	UK	Patients with suspected angina	1769	58 ± 10	56	30 ± 6	35	11	N/A	53	44
Xie_2018	Retrospective analysis of multinational CONFIRM registry	USA	Without known CAD	5039	N/A	N/A	N/A	54	18	54	21	29

Abbreviations: BMI, body mass index, CAD, coronary artery disease, N/A, not applicable.

**Table 2.** CAD-RADS score and outcome assessment in each study.

Name of cohort	CT scanner	CAD-RAD 0, %	CAD-RAD 1, %	CAD-RAD 2, %	CAD-RAD 3, %	CAD-RAD 4a, %	CAD-RAD 4b, %	CAD-RAD 5, %	Definition of adverse events	follow up duration	No. of events observed	Covariable s in multivariate analysis
Altay_2021	64-row CT	25	23	20	19	5	13	2	MACE: myocardial infarction, sudden cardiac death, bypass surgery, other cardiac surgeries, or stent implementation	8 years and 4 months (mean)	25	Age, gender, hypertension, diabetes mellitus, smoking, and family history
Bittner_2020	N/A	34	32	20	8	5	1 (4b+5)		Composite of death from any cause, myocardial infarction, or hospitalization for unstable angina	25 months (median)	115	ASCVD risk score
Finck_2019	64-row CT (both single source and dual source)	28	15	29	21	5	1	1	Composite of cardiac death or nonfatal myocardial infarction.	Median 10.0 years (IQR: 8.1 to 11.2 years)	58	Adjusted for Morise score
Huang_2021	Four CT systems	46	3.4	32.5	10.5	4.9	2.8 (4b+5)		All-cause mortality	4.3±2.1 years (median)	540	Patient age and sex
Johnson_2019	64-row CT	N/A	N/A	N/A	N/A	N/A	N/A	N/A	All-cause mortality	9 years (median)	380	N/A
Lee_2021	64-row SSCT or DSCT	50	20	14	7	8 (4a+b)		2	Composite of cardiac death, nonfatal myocardial infarction, and hospitalization for unstable angina	31.5 (7–55) months, median	103	Clinical risk factors
Nam_2019	DSCT or 64-row SSCT	23.5	19.7	18.2	18.6	15.4	2.2	2.4	Cardiovascular death, nonfatal myocardial infarction, and unstable angina requiring hospitalization or revascularization	3.36 ± 1.71 years (mean)	67	Clinical risk factors plus CAD extent classification
Park_2021	64-row SSCT or DSCT	55.8	14.2	14.5	7.4	5.5	2.1	6.5	Composite of cardiac death, MI, unstable angina, revascularization either by PCI or CABG, and heart failure required rehospitalization	2.13 years (median)	101	Clinical risk factors plus extent of CAD
Senoner_2020	64-slice CTA or 128-slice dual-source CTA	35.0	17.7	17.9	6	N/A	N/A	N/A	All-cause mortality or composite endpoint (fatal and nonfatal MACE)	Mean 10.55 ± 1.98 years (range, 6.1-12.8).	25 for CV mortality; 57 for MACE	Age ≥75 y, cigarette smoking, art. HT, DM.
van den Hoogen_2020_DM	≥64-slice CT scanners	29 (0 + 1)		51 (2 + 3)		20 (4 + 5)			Composite of all-cause death and nonfatal MI	5.1 (2.2–6.2) years (median)	95	age, sex, BMI, current smoking status, beta blockers, statins
van den Hoogen_2020_non_DM	≥64-slice CT scanners	37 (0 + 1)		48 (2 + 3)		15 (4 + 5)			Composite of all-cause death and non-fatal MI	5.1 (2.2–6.2) years (median)	60	Age, hypercholesterolemia, current smoking status, statins
van Rosendael_2019	64-slice or 320-slice CT scanner	53.9 (0 + 1)		40.6 (2 + 3)		5.5 (4 + 5)			all-cause mortality or non-fatal MI	3.6 ± 2.8 years	130	Age, sex, hypertension, hypercholesterolemia, diabetes mellitus, smoking, family history of CAD
Williams_2020	N/A	0	6	16	13	27	26	31	Occurrence of coronary heart disease death or nonfatal MI	4.7 (4.0 to 5.7) years (median)	41	N/A
Xie_2018	≥64-slice CT scanners	33	12	12	15	20	4	4	Composite outcome of death or nonfatal MI	5 years	314 deaths and 457 non-fatal MI	Age, sex, hypertension, diabetes, hyperlipidemia, smoking, and chest pain characteristics

Abbreviations: ASCVD, Atherosclerotic cardiovascular disease; BMI, body mass index; CABG, Coronary artery bypass graft surgery; CAD-RADS, Coronary Artery Disease-Reporting and Data System; CT, computed tomography; CTA, computed tomography angiography; CV, cardiovascular; DSCT, Dual source computed tomography; DM, diabetes mellitus; HT, hypertension; IQR, interquartile range; MACE, major adverse cardiovascular event; MI, myocardial infarction; N/A, not applicable; PCI, Percutaneous coronary intervention; SSCT, Single source computed tomography.

### Prognostic capability of CAD-RADS categories in patients with suspected CAD

**Figure 2.**
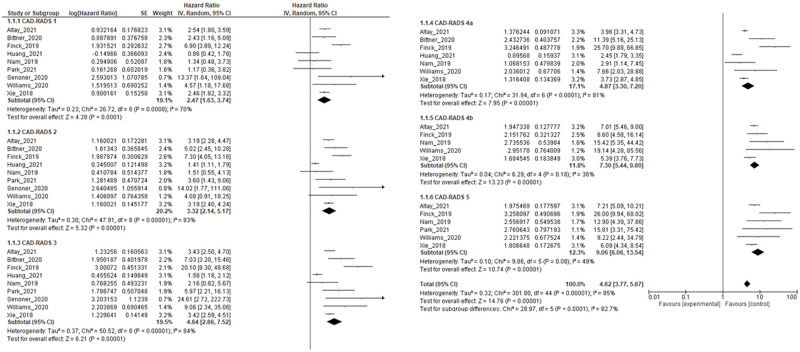
Pooled hazard ratio (HR) for each Coronary Artery Disease-Reporting and Data System (CAD-RADS) category in predicting adverse events. This figure presents the pooled HR for each CAD-RADS category, with CAD-RADS=0 serving as the reference.

During the follow-up period, adverse events were detected in 2,536 patients, which corresponds to a prevalence of 6.7%. The pooled HR demonstrated a slight increase in CAD-RADS 1 (HR, 2.47; 95% confidence interval [CI], 1.63-3.74; *P* < .001; *I*^2^ = 70%; heterogeneity *P* < .001) and CAD-RADS 2 (HR, 3.32; 95% CI, 2.14-5.17; *P* < 0.001; *I*^2^ = 83%; heterogeneity *P* < .001). In CAD-RADS 3 (HR, 4.64; 95% CI, 2.86-7.52; *P* < .001; *I*^2^ = 84%; heterogeneity *P* < .001) and CAD-RADS 4a (HR, 4.87; 95% CI, 3.30-7.20; *P* < 0.001; *I*^2^ = 81%; heterogeneity *P* < .001), the HR gradually increased. The pooled HR seemed to increase with CAD-RADS 4b compared with 4a (7.30; 95% CI, 5.44-9.80; *P* < .001; *I*^2^ = 36%; heterogeneity *P* = .18) although this difference could not be shown to be statistically significant. The pooled HR for CAD-RADS 5 was the highest in all categories (9.06; 95% CI, 6.06-13.54; *P* < .001; *I*^2^ = 49%; heterogeneity *P* = .08) (Figure 2).

**Figure 3.**
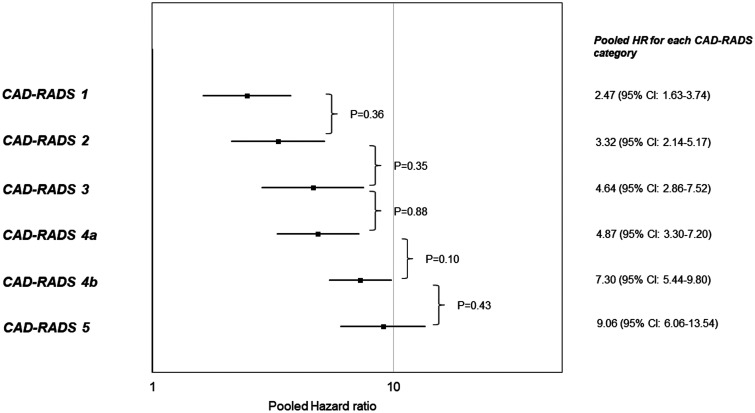
Comparative analysis of pooled hazard ratios (HR) for each Coronary Artery Disease-Reporting and Data System (CAD-RADS) category for all events. The HRs exhibited a gradual increase as the CAD-RADS category increased, with a rise observed between CAD-RADS 4a and 4b (*P* = .10 according to the chi-square test).

A progressive increase in pooled HRs was observed as the categories increased, and Figure 3 shows significant HR differences by adjacent CAD-RADS category. When CAD-RADS 4a and 4b HRs were 4.87 (95% CI, 3.30-7.20) and 7.30 (95% CI, 5.44-9.80) were compared, an increasing trend was observed, although this did not reach statistical significance (*P* = .10). No statistically significant differences were found among the other categories as well (*P* = 0.35-0.88).

**Figure 4.**
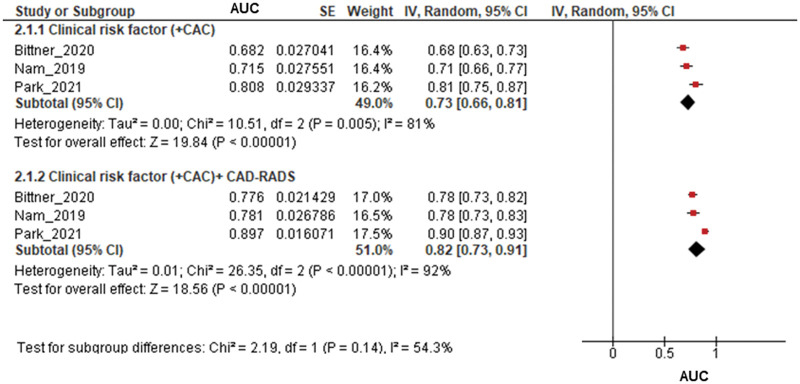
Incremental prognostic value of Coronary Artery Disease-Reporting and Data System (CAD-RADS) in addition to clinical risk factors. The addition of CAD-RADS to the clinical risk factors resulted in an area under the curve (AUC) of 0.82 (95% confidence interval, 0.73-0.91) for predicting major cardiac adverse events.

Figure 4 compares the AUC in predicting events between the traditional clinical risk factor (pooled AUC of 0.73 [95% CI, 0.66-0.81; *I*^2^ = 92%; *P* < .001; *I*^2^ = 81%; *P* for heterogeneity = .005] and CAC) and with the addition of CAD-RADS to the traditional clinical risk factor and CAC) (pooled AUC of 0.82 [95% CI, 0.73-0.91; *P* < .001; *I*^2^ = 92%; *P* for heterogeneity < .001]). Although an increase was noted, this difference was not statistically significant (*P* = .14).

### Prognostic ability of CAD-RADS classified by endpoints

**Figure 5.**
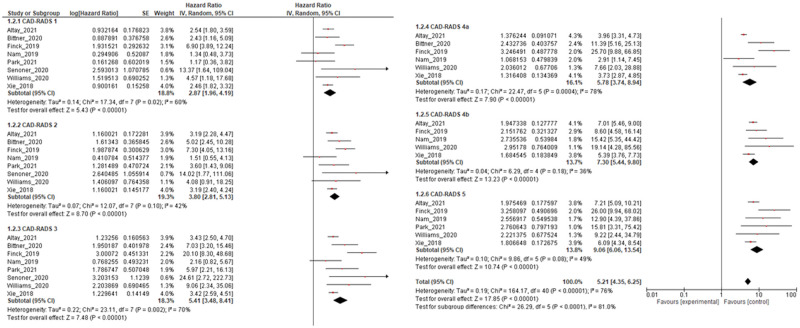
Forest plot of hazard radio (HR) of Coronary Artery Disease-Reporting and Data System (CAD-RADS) scores on major adverse cardiovascular event prediction.

**Figure 6.**
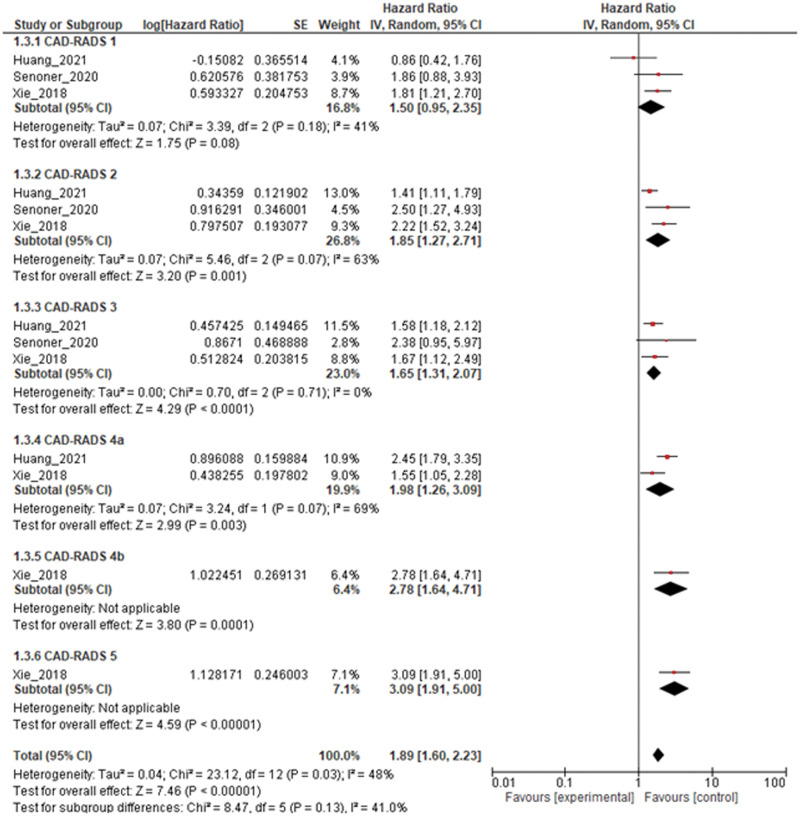
Forest plot of hazard radio hazard ratio (HR) of Coronary Artery Disease-Reporting and Data System (CAD-RADS) scores on all-cause mortality prediction.

We assessed the prognostic potential of CAD-RADS for 2 endpoints: all-cause mortality and MACE. Only 3 studies investigated the HR of CAD-RADS for predicting all-cause mortality. In contrast, there were 9 studies that presented HRs for MACE. Two studies presented HRs for both all-cause mortality and MACE. Figure 5 shows a forest plot showing the pooled HR of the CAD-RADS score for MACE, which was a significant prognostic factor in all CAD-RADS 1 through 5 categories. On the other hand, the CAD-RADS forest plot for the prediction of all-cause mortality, shown in Figure 6, was a significant prognostic predictor in all categories except CAD-RADS 1.

### Results of the risk of bias assessment

The quality of studies assessed for risk of bias is summarized in Table S2: of the 13 studies, 8 (62%) were rated as high quality (>80% on a quality scale), 5 (38%) were rated as moderate quality (between 50% and 80% on a quality scale), and no studies were rated as low quality (<50% on a scale). Analysis of the pooled HR of CAD-RADS scores by the fixed effect model for sensitivity analysis showed similar results to the random effect model (Figure S1). The Begg test detected a significant possible publication bias in the pooled HRs of CAD-RADS, with a Kendall's tau value of 0.2646 and a *P* value of .0101 (Figure S2).

## Discussion

The primary findings of this study can be summarized as follows: First, CAD-RADS provides useful prognostic information for patients with suspected CAD, with the HR increasing as the score increases. Second, the HR increased from CAD-RADS 4a to 4b and showed an increasing trend (*P* = 0.10). Finally, when combined with traditional clinical risk factors and CAC, CAD-RADS was found to predict adverse events with a high accuracy (AUC = 0.82).

CAD-RADS is a tool that not only objectively evaluates the severity of coronary CTA, but also provides recommendations for postdiagnosis treatment. It offers specific guidelines for appropriate care based on noninvasive imaging results, and its effective use has been shown to reduce unnecessary invasive coronary angiographies, particularly for CAD-RADS 3.

Prognostic stratification is crucial for appropriate care of CAD patients. However, many of these studies are retrospective analyses or case studies, and as such, they may be subject to biases. For example, in MACE prediction, CAD-RADS 4a, 4b, and 5 are consistent in a positive direction in all studies (Figure 5). However, in CAD-RADS 1, 2, and 3, the HR of CAD-RADS is not significant in multiple studies. However, the meta-analysis confirmed significant prognostic power in CAD-RADS 1, 2, and 3. We believe the significance of this meta-analysis is that it showed consistent usefulness across all CAD-RADS categories.

A recent meta-analysis of the prognostic potential of CAD-RADS was conducted that calculated a pooled HR for CAD-RADS 4A and 4B of 7.29 and 6.27, respectively. This seems contrary to the expected worse prognosis with more severe lesions (ie, left main coronary artery lesions) categorized as CAD-RADS 4B (ie, left main coronary artery lesions). In the present study, the risk of adverse events gradually increased with increasing CAD-RADS. The pooled HRs of CAD-RADS 4A and 4B were 3.9 and 7.25, respectively, which is more consistent with the expected pathophysiology, although we have not directly compared our analysis to one previously published. However, 1 reason for this difference may be the noninclusion of the study by Nam et al in the previous meta-analysis that may have caused the overestimation of the HR for CAD-RADS 4a.

In the present study, the risk of MACE increased progressively with increasing CAD-RADS, as expected. Interestingly, however, CAD-RADS 4B seemed to show an increase in hazard ratio compared to 4A, albeit one that could not be shown to be statistically significant. This is consistent with the pathophysiology of CAD in which 4B patients are classified as higher risk. CAD-RADS 4B is designated for patients presenting with left main coronary artery lesions or severe 3-vessel lesions. CAD-RADS 4B category has been excluded from the ISCHEMIA study,^29^ particularly in cases involving critical ischemia resulting from left main coronary artery lesions. There is considerable ongoing debate regarding the optimal medical approach, encompassing considerations of pharmacotherapy versus invasive revascularization. The deterioration in prognosis observed among patients classified as CAD-RADS 4b not only reflects the severity of the disease itself, but also raises concerns about the adequacy of current medical care. It is crucial to conduct further research to identify optimal medical care strategies for this specific patient population. Risk stratification using the CAD-RADS score may prove valuable in guiding patient selection and improving outcomes.

In recent years, many studies using artificial intelligence (AI) in the field of diagnostic imaging have garnered significant attention.^30^ The use of AI may be helpful for prognostic estimation by scoring a wide range of coronary atherosclerosis features that are not evaluated by CAD-RADS, as has been reported to be useful in previous studies.^13^ Although the present study only evaluated the effectiveness of CAD-RADS alone, better results may be obtained by considering risk factors other than imaging. For example, the CAC score is a straightforward measure that can be used to predict the prognosis of MACE in patients with chest pain. A CAC score of 0 indicates an annual risk of 0.8% for MACE, which is remarkably low, according to a meta-analysis.^31^ Currently, no machine learning studies have been conducted that combine coronary risk factors and coronary CT findings. Future research is anticipated to incorporate clinical risk factors, imaging findings, and AI-based risk stratification for more effective therapeutic care.

## Study limitations

The study has several limitations that should be acknowledged. First, the articles incorporated in the meta-analysis composed studies with relatively restricted sample sizes, predominantly retrospective designs, and retrospective analyses of extensive studies that exhibited substantial heterogeneity in the analysis outcomes. Second, each study provided distinct definitions for events. Numerous studies employed composite endpoints, and future investigations should evaluate HRs specifically for purely cardiac endpoints. Third, when interpreting the prognostic data, it should be noted that CAD-RADS provides treatment recommendations based on the findings, but this does not necessarily imply that the recommended treatment strategy was followed. Fourth, CAD-RADS has recently been updated but was not evaluated in this study; CAD-RADS 2.0 could provide more detailed information on myocardial ischemia and coronary plaque, which may help to better predict outcomes.^32^

## Conclusions

The CAD-RADS category imparts information on the CAD severity and shows incremental increase in HR for MACE with progressively higher categories, especially beyond CAD-RADS 4b. The risk stratification of CAD when evaluated in combination with conventional clinical risk factors and coronary artery calcification offers a high predictive capacity for MACE.

## Supplementary Material

umae007_Supplementary_Data

## Data Availability

The data that support the findings of this study are available from the corresponding author upon reasonable request.
