# Supplementary material for: Prognostic significance of CAD-RADS for patients with suspected coronary artery disease: A systematic review and meta-analysis
Source: Radiol Adv. 2024 Apr 1;1(1):umae007. doi: 10.1093/radadv/umae007 (PMC12429183; doi:10.1093/radadv/umae007)
Supplement: umae007_Supplementary_Data [file umae007_Supplementary_Data.zip › R2_Supplemental material.docx]

**Table S1**

*PubMed 179*

*#1 (coronary artery disease [title] OR stable angina pectoris [title] OR CAD [title] OR stable AP [title] OR myocardial infarction [title] OR MI [title])*

*#2 (coronary computed tomography [title] OR coronary CTA [title])*

*#3 (CAD-RADS [title] OR Coronary Artery Disease Reporting and Data System [title])*

*#4 #1 AND #2 AND #3*

*WOS 141*

*#1 TI=(coronary artery disease [title] OR stable angina pectoris [title] OR CAD [title] OR stable AP [title] OR myocardial infarction [title] OR MI [title])*

*#2 TS=(coronary computed tomography [title] OR coronary CTA [title])*

*#3 TS=(CAD-RADS [title] OR Coronary Artery Disease Reporting and Data System [title])*

*#4 #1 AND #2 AND #3*

*Cochrane 29*

*#1 coronary artery disease:ti OR stable angina pectoris:ti OR CAD:ti OR stable AP:ti OR myocardial infarction:ti OR MI:ti*

*#2 coronary computed tomography:ti OR coronary CTA:ti*

*#3 CAD-RADS:ti OR Coronary Artery Disease Reporting and Data System:ti*

*EMBASE*

*QUICK SEARCH:303*

*TITLE: (coronary artery disease OR stable angina pectoris OR CAD OR stable AP OR myocardial infarction OR MI)*

*AND (coronary computed tomography OR coronary CTA)*

*AND (CAD-RADS OR Coronary Artery Disease Reporting and Data System)*

**Table S2**

NEWCASTLE - OTTAWA QUALITY ASSESSMENT SCALE CASE CONTROL STUDIES

| **Study** | **Selection**  **(Max=4)** | **Comparability**  **(Max=2)** | **Exposure**  **(Max=3)** | **Total Score** |
| --- | --- | --- | --- | --- |
| Altay_2021 | 3 | 2 | 3 | 8 |
| Bittner_2020 | 4 | 2 | 3 | 9 |
| Finck_2019 | 4 | 2 | 3 | 9 |
| Huang_2021 | 3 | 2 | 2 | 7 |
| Johnson_2019 | 3 | 2 | 2 | 7 |
| Lee_2021 | 3 | 2 | 3 | 8 |
| Nam_2019 | 3 | 2 | 2 | 7 |
| Park_2021 | 3 | 2 | 2 | 7 |
| Senoner_2020 | 3 | 2 | 2 | 7 |
| van den Hoogen_2020 | 4 | 2 | 3 | 9 |
| van Rosendael_2019 | 4 | 2 | 3 | 9 |
| Williams_2020 | 4 | 2 | 3 | 9 |
| Xie_2018 | 4 | 2 | 3 | 9 |
