# Supplementary material for: Prognostic significance of CAD-RADS for patients with suspected coronary artery disease: A systematic review and meta-analysis
Source: Radiol Adv. 2024 Apr 1;1(1):umae007. doi: 10.1093/radadv/umae007 (PMC12429183; doi:10.1093/radadv/umae007)
Supplement: umae007_Supplementary_Data [file umae007_Supplementary_Data.zip › R2_Supplemental Figure.docx]

**Figure S1. Forrest plot of HR for CAD-RADS scores with fixed effect model.**

**
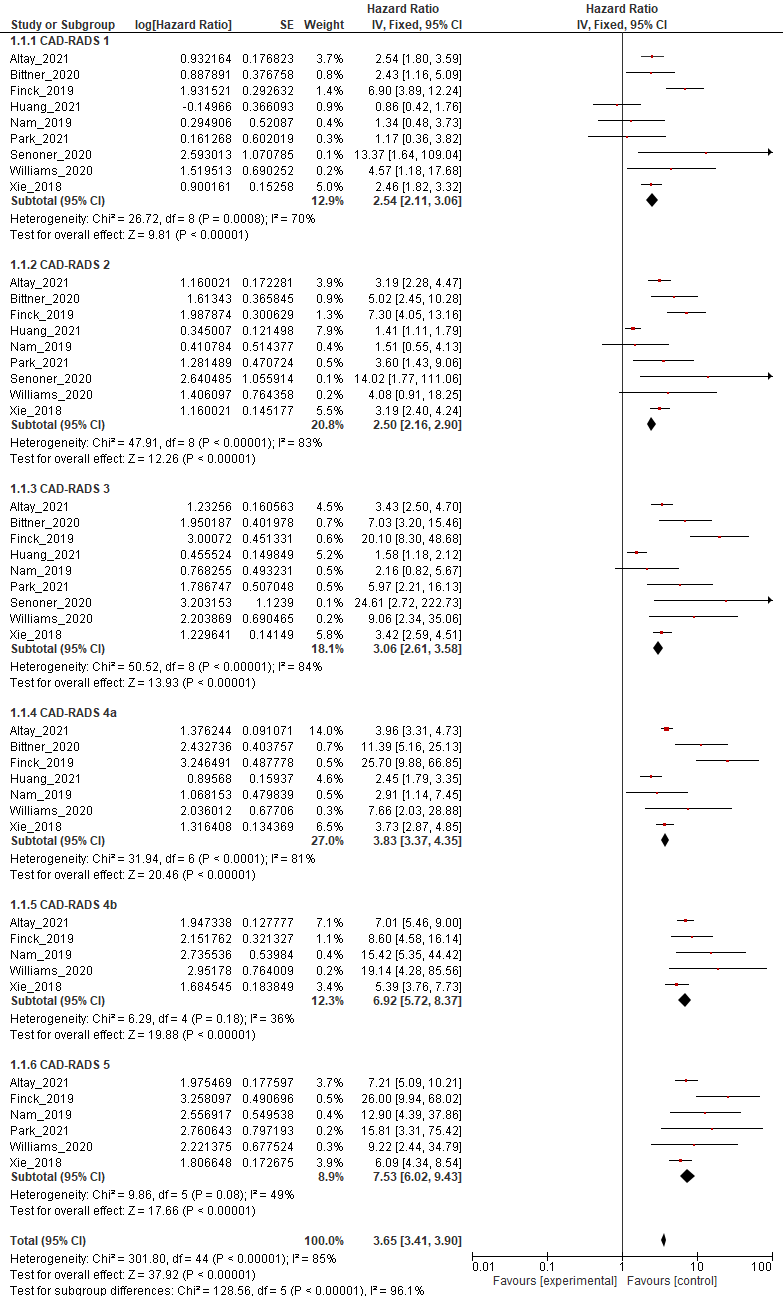
**

**Figure S2**


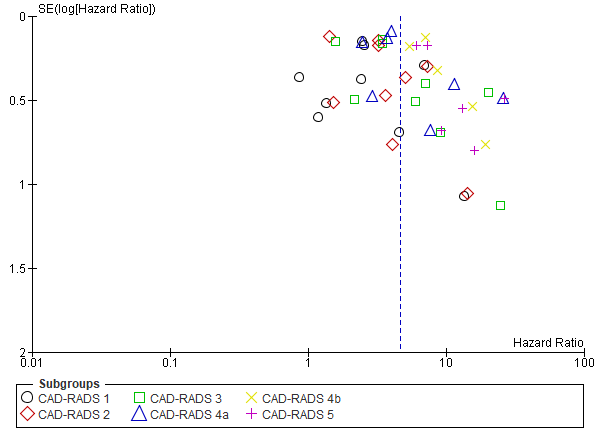


The funnel plot displays the HR of CAD-RADS and serves as a graphical representation of potential publication bias. The Rank Correlation Test for Funnel Plot Asymmetry demonstrated a Kendall's tau value of 0.2646, p = 0.0101, which suggests possible publication bias.
